# Headache and non-headache symptoms provoked by nitroglycerin in migraineurs: A human pharmacological triggering study

**DOI:** 10.1177/0333102420910114

**Published:** 2020-03-12

**Authors:** Nazia Karsan, Pyari R Bose, Charlotte Thompson, Jayde Newman, Peter J Goadsby

**Affiliations:** 1 Headache Group, Department of Basic and Clinical Neuroscience, Institute of Psychiatry, Psychology and Neuroscience, King's College London, London, UK; 2 NIHR-Wellcome Trust King's Clinical Research Facility, King's College Hospital, London, UK; 3 SLaM Biomedical Research Centre, King's College London, London, UK

**Keywords:** Migraine, premonitory, headache, nitroglycerin, trigger, provocation, migraine triggering

## Abstract

**Background:**

Studying a spontaneous migraine attack is challenging, particularly the earliest components. Nitroglycerin is a potent, reliable and reproducible migraine trigger of the entirety of the migraine attack, making its use experimentally attractive.

**Methods:**

Fifty-three subjects with migraine with a history of spontaneous premonitory symptoms were exposed to a 0.5 mcg/kg/min nitroglycerin infusion. Eighty-three percent (n = 44) developed typical premonitory and headache symptomatology. Fifty-seven percent (n = 25) were invited back to further study visits, during which they were re-exposed to nitroglycerin or placebo infusion in a double-blind randomised design. The phenotype of premonitory symptoms and headache was captured and compared to spontaneous attacks and between triggered attacks using agreement analysis.

**Results:**

More premonitory symptoms were triggered with nitroglycerin than placebo (mean symptom difference = 4, *t*_20_ = 7.06, *p* < 0.001). The agreement in triggering for the most commonly reported premonitory symptoms (concentration difficulty and tiredness) was >66%. The retriggering agreement for all but one premonitory symptom was >60%. The agreement in timing to onset of premonitory symptoms was reliable across two triggered attacks. The agreement with spontaneous attacks and between attacks for headache and its associated symptoms, including laterality, was less reliable.

**Conclusions:**

Nitroglycerin can reliably and reproducibly provoke premonitory symptomatology associated with migraine. This forms an ideal model to study the earliest manifestations of migraine attacks.

## Introduction

Non-headache symptomatology associated with migraine has been noted since at least the 19th century (1), while systematic studies of the prevalence and phenotype have only really emerged over the last 30 years (2,3). Understanding the non-painful symptoms that can accompany migraine headache is vital to understanding of the neurobiology of the disorder and to appreciating more completely the disability associated with each attack.

Non-headache symptomatology associated with migraine can start up to 3 days prior to pain onset in some individuals, can predict pain onset (4), and can also persist following headache resolution and impair return to normal function (5). Understanding the neurobiological basis of the early and predictive premonitory symptoms, a terminology used herein over prodrome in ICHD 3 (6), in line with previous and current literature is vital to furthering knowledge about how and where a migraine attack starts within the brain. This knowledge could help in the identification of novel therapeutic targets for attack abortion prior to pain onset, which would be an attractive therapeutic avenue for both patients and physicians alike.

Capturing the entirety of a migraine attack to appreciate these symptoms fully and perform detailed phenotyping prospectively, as well studying spontaneous headache experimentally, are challenging issues. The early and often unpredictable onset of premonitory symptoms, their broad and heterogeneous phenotype, overlap with triggers (7,8), and the usually long duration of a migraine attack make experimental observation of the entire attack difficult.

Human migraine pharmacological provocation models have been designed to address these issues, with reliable provocation of a migraine attack in a shorter and more compressed time frame compared to spontaneous attacks (9). The best-established pharmacological migraine trigger agent is intravenous nitroglycerin (10). Nitroglycerin administered intravenously at 0.5 mcg/kg/min over 20 minutes is able to provoke migraine headache in up to 83% of sufferers (11–13) and has been shown to be able to trigger premonitory symptoms in some sufferers (11). This study looking at premonitory symptoms triggered with NTG amongst migraineurs also exposed healthy controls to nitroglycerin, but unfortunately data from healthy controls regarding the development of non-painful symptomatology following nitroglycerin exposure is not available (11). The ability of nitroglycerin to provoke premonitory symptoms has been exploited for the purpose of imaging the earliest premonitory symptoms using functional neuroimaging (14).

## Objectives

This study aimed to use the nitroglycerin provocation model to examine non-headache symptomatology associated with the migraine attack, as well as headache, to compare triggered attacks to spontaneous attacks, and to compare serial triggered attacks to each other. This was performed with a view to assessing the reliability and reproducibility of the model in provoking migraine symptoms in a systematic fashion, to allow further assessment of its role in experimental migraine research and in therefore furthering understanding of migraine neurobiology. Whilst this methodology has been used before, repeated exposure to nitroglycerin is less well documented in the literature, as is the detailed phenotype of nitroglycerin-triggered attacks. The work was reported in preliminary form at the 19th Congress of the International Headache Society (Dublin, 5–8 September, 2019) (8).

## Methods

### Subjects

Subjects with migraine were identified through online advertisements, bulletins and patient group advertising through the Migraine Trust, a newspaper advertisement, advertising around the university for staff and student volunteers and through local and national headache clinics. The inclusion criteria for the study included a diagnosis of migraine with or without aura, as per ICHD-3 beta, which was in use at the time of the study (15), with up to 22 headache days a month, a history of spontaneous premonitory symptoms with attacks, and no contraindications to study participation and/or nitroglycerin exposure. Use of any single agent oral preventive therapy for migraine was allowed. Exclusion criteria included medication overuse (16), use of more than one oral preventive agent for migraine, or the use of neuromodulatory devices, or both, and onabotulinum toxin type A and/or greater occipital nerve injections within the last 3 months. Illicit drug use and excess alcohol and tobacco consumption were also excluded.

Given the generally poor ability of NTG to trigger aura (12,17,18), we did not anticipate that aura would be triggered in the study and act as a potential confound and was therefore allowed in the study. Additionally, we experienced difficulty in recruitment to identify infrequent episodic migraine without aura. Given the definition of chronic migraine is simply determined by at least half the month involving headache (6), and biologically the difference between a migraineur with headache 14 days and 15 days a month is likely equivocal, we decided to include up to 22 headache days a month to aid recruitment. For similar recruitment reasons, and for interest, we also decided to include subjects on single agent preventive therapy, to assess if there was an impact of preventive use on nitroglycerin triggering.

Recruitment was completed from February 2015 to July 2017.

### Ethical approval

This study using human participants was approved by the Camden and King's Cross Research Ethics Committee in February 2015 (14/LO/2241). The study was also approved by King's College Hospital Research and Development Committee. All subjects enrolled in the study gave informed written consent for participation, according to the Declaration of Helsinki.

### Sample size

We aimed to study sufficient subjects (n = 53) to exceed the current literature number of subjects exposed to nitroglycerin (n = 44) (11). We therefore aimed to expose at least 50 subjects to nitroglycerin. Given one of the objectives of the study was to compare reproducibility of symptoms across nitroglycerin exposures, we aimed to have at least 70% of recruited subjects trigger premonitory symptoms and headache and therefore have more than one exposure to nitroglycerin (n = 35) and complete at least two visits and ideally at least half (n = 25) also to have exposure to placebo for comparison. The study was challenging to conduct, owing to the high screening to eligibility for recruitment ratio, unwillingness for serial exposures to nitroglycerin, and some loss of follow up of subjects when trying to schedule further visits. We therefore chose to keep the study recruitment number at around 50 and compromise on reducing sample size throughout the study with each visit.

### Screening

Three hundred and fifty subjects made email or telephone contact with the study team and were pre-screened for eligibility. Of these 350 subjects, 53 (15%) met eligibility criteria and agreed to attend a screening visit. There was a large pre-screening failure rate, mostly owing to too-frequent headache, preventive medication use and other excluded medications (see Table 1).

**Table 1.** Reasons for pre-screen failures in the study.

Reason for screen failure	n	% of screen failures
Too frequent headache	147	49
Too many migraine preventive treatments	52	18
Other excluded medications	36	12
Medical/psychiatric comorbidity	21	7
Medication overuse	17	6
Unwilling to attend multiple study visits/be triggered	8	3
Lives too far and unable to commute to study visits	5	2
Age outside of study parameters	5	2
Contraindication to 3Tesla MRI scanning	4	1
Female subjects planning pregnancy	2	0.6

All study visits were performed within the Clinical Research Facility at King's College Hospital.

The screening visit involved written consent for study participation, followed by detailed phenotyping of spontaneous migraine attacks, triggers, medication history and ensuring no medical or pharmaceutical contraindications to any of the study drugs, including nitroglycerin and acute migraine treatments used to treat pain in the study: intravenous aspirin and subcutaneous sumatriptan. An appropriate cardiovascular and neurological examination was performed. An ECG was performed to exclude cardiac contraindications to nitroglycerin or triptan exposure. The spontaneous migraine attacks were phenotyped using the same symptom questionnaire used for symptom capture during the triggered attack (see Table 2). A MIDAS score was completed for each participant (19).

**Table 2.** Detailed phenotyping symptom checklist used for symptom capture in the study.

Premonitory symptoms	Thirst □ Elation □ Visual blurring □ Cravings □ Depression □ Neck stiffness □ Yawning □ Irritability □ Photophobia □ Fatigue □ Concentration difficulty □ Phonophobia □ Urinary frequency □ Speech difficulty □ Gi discomfort □ Movement sensitivity □
Headache features	Side preference: Right □ Left □ Bilateral □ Conjunctival injection □ Photophobia □ Conjunctival tearing □ Phonophobia □ Ptosis □ Allodynia □ Eyelid swelling □ Neck stiffness □ Nasal stuffiness □ Dizziness □ Rhinorrhoea □ Visual blurring □ Aural fullness □ Osmophobia □ Pupillary change □ Facial flushing □ Facial sweating □ Taste disturbance □

### Triggering

Following the history and examination, each subject was exposed to a 0.5 mcg/kg/min nitroglycerin infusion over 20 minutes, to identify those subjects who developed premonitory symptoms and headache. Subjects were symptomatically and haemodynamically assessed with blood pressure, heart rate and oxygen saturation monitoring before the infusion and at 5-minute intervals during the infusion, with questioning regarding the evolution of any headache, its site, severity, phenotype and the presence of any other symptoms, including typical premonitory symptoms. Questioning continued at 15-minute intervals following the infusion until the time of headache resolution following treatment. The complete symptom questionnaire is shown in Table 2. The answers to the premonitory symptom question presence were binary (yes/no), therefore any reported or observed change from baseline was considered positive.

**Figure 1.**
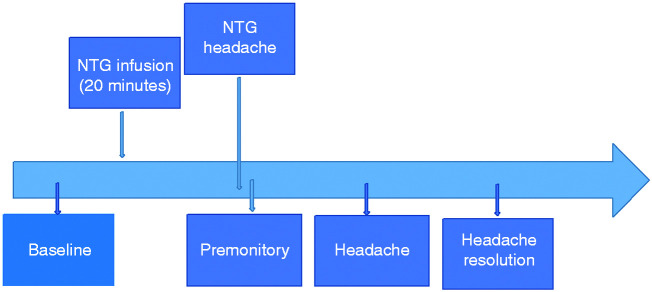
Summary of visit timeline for a nitroglycerin-triggered visit.

A premonitory symptom was defined as any symptom that the patient experienced following nitroglycerin infusion in the absence of migraine headache, which was typical for a symptom they would experience prior to a spontaneous migraine headache. The premonitory phase was defined as the presence of at least three typical symptoms for the patient occurring prior to delayed migraine headache. Headache was defined as per the modified experimental migraine criteria for experimental studies (20). See Figure 1 for a summary of the visit timeline.

Nitroglycerin itself is a drug agent with vasodilatory actions, often exploited in cardiology use for coronary vasodilation. The drug can cause side effects: notably reported are headache, tachycardia, hypotension, nausea and vomiting and asthenia (21). While perhaps asthenia and nausea may be mistaken as premonitory symptoms, the drug half life is short (minutes) (22), and vasodilatory effects are thought to peak at a similar time, and possibly last for no more than 45 minutes within the brain (23,24). These side effects typically come on soon after administration, particularly in the case of intravenous administration, and usually resolve within 5–10 minutes of administration. We would therefore considered that with the design of the study, drug effects, if any, would be minimal or absent at the time defined as the premonitory phase of migraine for each subject.

Following the screening visit, further study visits were conducted in a double-blind randomised fashion, with crossover of re-triggering with nitroglycerin or placebo. Of the subjects who triggered both premonitory symptoms and headache at the screening visit following nitroglycerin infusion, 25 were agreeable to re-attend for further visits. All following visits occurred with at least 2-week intervals and were performed in the morning to keep the timing of nitroglycerin exposure consistent throughout the study. Randomisation for which trigger agent was administered at subsequent visits was performed using the *RAND random number generator in Microsoft Excel. All drug preparation and administration at these visits was performed by an unblinded investigator and out of sight of the subject. For the placebo visit, the infusion (clear and colourless) was administered in the same way as nitroglycerin and in the same volume over 20 minutes, and the symptomatic and haemodynamic questioning was identical to the triggered visit, which was as for the screening visit. Subjects had to be completely pain-free, and free of any migraine symptomatology (premonitory symptoms, headache and postdrome symptoms), as well as of any acute migraine abortive medication for at least 12 hours prior to a visit. While nitrate tolerance is described in cardiology practice (25), it has never been studied in pharmacological migraine provocation studies, and therefore in line with other serial nitroglycerin triggering studies (11,14), we felt that a period of 2 weeks in between visits was appropriate. In addition, none of the subjects in the study used long-acting triptans or abortive medications (Table 3) that we would expect to continue to have an effect on the threshold of nitroglycerin triggering.

**Table 3.** Summary of enrolled subjects demographics.

*Subject demographic*	
Age (years, mean ± SD)	36 ± 9
Gender (M/F)	9 M 44 F
Headache days at baseline (days/month, median ± IQR)	8(4.5–12)
Diagnosis (EMA, EMO, CM)	27 EMA 20 EMO 6 CM
Duration of disease (years, median ± IQR)	18 ± 13
MIDAS score (median ± IQR)	20(11–40)
Spontaneous laterality of headache (L/R/bilateral)	16 L 18 R 19 bilateral
Lifetime aura (yes/no)	32 Y 21 N
Active aura (aura within the last year, yes/no)	30 Y 23 N
Acute medications used for attacks	Paracetamol/NSAID combination 10 Triptan (sumatriptan/ rizatriptan/almotriptan) 25 Aspirin 4 NSAID alone 7 Paracetamol/codeine combination 5 Triptan/NSAID combination 2
Preventive use (yes/no)	16 Y 37 N

EMA: episodic migraine with aura; EMO: episodic migraine without aura; CM: chronic migraine; NSAID: non-steroidal anti-inflammatory.

For the placebo visit, a premonitory-like symptom was defined as a symptom typical for a patient prior to a spontaneous migraine attack, but without subsequent migraine headache developing.

**Figure 2.**
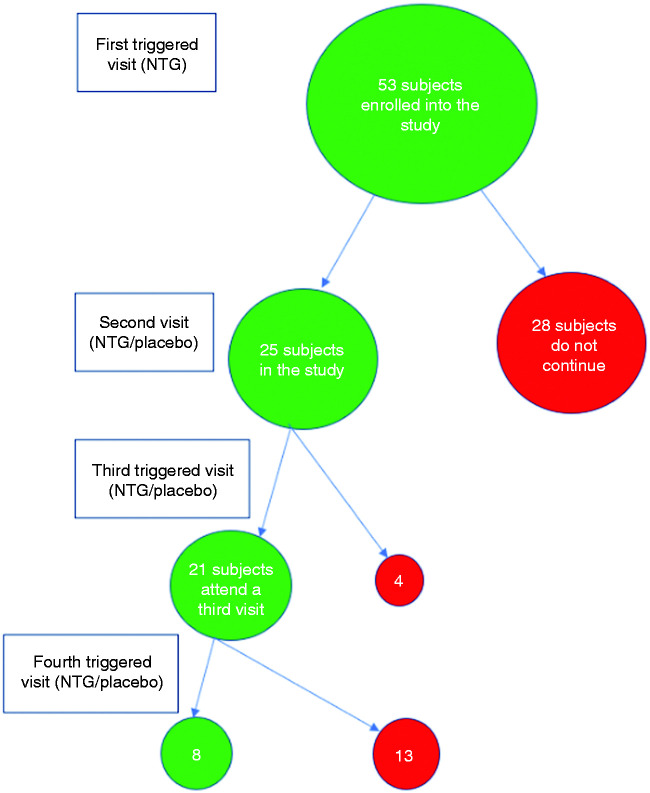
Subject numbers in each stage of the study.

Subject numbers throughout the study are summarised in Figure 2.

### Treatment of triggered attacks

All delayed migrainous headache that ensued following nitroglycerin infusion was treated as soon as it reached moderate-severe intensity, using either 1 g intravenous aspirin or 6 mg subcutaneous sumatriptan, based on whether the subject usually responded to a non-steroidal anti-inflammatory drug (NSAID) or to a triptan, respectively. In the event of an inadequate treatment response, the alternative treatment could be offered for headache cessation. Headache freedom was necessary before a subject could be discharged from the Research Facility.

### Statistical analysis

Statistical analyses were performed using SPSS version 24 and STATA. Agreement analysis was performed using Cohen's kappa analysis (26), or intraclass correlation coefficient within SPSS or Krippendorff's alpha within STATA (27). Correlation analysis was performed using Pearson correlation in SPSS. Comparison of symptom numbers reported with NTG and placebo was performed using a paired *t*-test in SPSS. In all statistical analyses, where relevant, significant results are highlighted with an asterisk (*). When using dichotomous data, the Cohen's kappa coefficient value is not always reflective of the percentage agreement (28). For this reason, for this study, symptom reporting percentage agreement of 60% or more was considered moderate agreement and these results will also be highlighted, irrespective of the Cohen's kappa value. For the purpose of agreement reporting, >60% agreement, or kappa/Krippendorff's alpha >0.4, or both, was considered significant. For timing, a significant intraclass correlation coefficient (*p* < 0.05) was considered significant.

## Results

### Subject demographics

Of the 53 subjects – nine were male, 27 had migraine with aura, 20 had migraine without aura, 6 had chronic migraine, and 16 (30%) were on single agent preventive therapy. The majority (38%) were on a beta-blocker, with use of amitriptyline, topiramate, candesartan and pizotifen being less common. The age range of subjects was 18–50 years (mean 36 years), with up to 22 headache days per month (median 8 days, range 1–22 days). Subject demographics are summarised in Table 3.

Forty-four subjects developed delayed migrainous headache following the nitroglycerin infusion (83%). Of these, 52/53 (98%) had typical premonitory symptomatology preceding the headache. Of these 44, 33 met the study criteria to attend a second visit (75%), based on timeline to symptom development, treatment response of headache and willingness to re-attend for further study visits, but only 25 re-attended. Twenty-one attended three study visits, thus being exposed nitroglycerin twice, as well as placebo.

### Visit 1: Triggering rates

Of 53 subjects, 52 developed one or more premonitory symptoms. When assessing the correlation between baseline headache frequency and likelihood of triggering migraine headache with NTG, Pearson correlation coefficient was 0.296, *p* = 0.031; suggesting a weak positive correlation between the number of headache days at baseline and the likelihood of triggering, without accounting for other factors that may impact on the likelihood of triggering with NTG.

Typical aura symptoms were triggered in 7 of the 53 subjects at visit 1 (13%). Four subjects had the same symptoms again on a second NTG exposure. The aura phenotypes were visual aura (one at visit 1 was not followed by delayed migraine), hemisensory, hemimotor, and Alice in Wonderland syndrome.

### Visit 1: Timing of attacks

**Figure 3.**
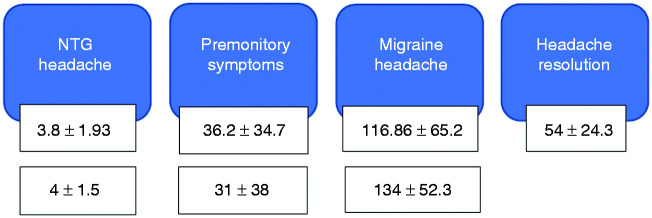
Summary of the timeline to development of symptoms on the first (top row) and second nitroglycerin-triggered visits (second row) (mean + /− standard deviation in minutes).

The timing to development of various symptoms is shown in Figure 3.

All 53 subjects (100%) developed NTG-headache within 10 minutes of the start of the nitroglycerin infusion (range 1–10 minutes, median 4 minutes).

Fifty-two subjects (98%) developed at least one premonitory symptom following the infusion within 4–155 minutes following the start (median 23 minutes). The range of premonitory symptoms triggered in these subjects was 1–7.

Forty-four subjects (83%) developed migrainous headache following the NTG infusion (range 20–278 minutes following infusion start, median 107 minutes).

The percentage agreement and agreement analysis between spontaneous and first triggered attack for premonitory and headache symptoms is shown in Table 4. All premonitory symptoms apart from mood change and photophobia displayed moderate to good agreement in reporting between spontaneous and first triggered attack.

**Table 4.** Agreement analysis between spontaneous and first NTG-triggered attack for premonitory symptoms and associated headache symptoms.

Symptom	Cohen's kappa coefficient	Percentage agreement between spontaneous and first triggered attack (%)	*p*-value
Premonitory			
Tiredness	0.51	85%* (45/53)	<0.001
Neck stiffness	0.4	70%* (37/53)	0.006
Yawning	0.3	64%* (34/53)	0.028
Mood change	0.075	47% (25/53)	0.468
Thirst	0.4	72%* (32/53)	0.004
Concentration change	0.2	66%* (35/53)	0.117
Photophobia	0.14	51% (27/53)	0.177
Headache			
Photophobia	0.5	91%* (40/44)	<0.001
Phonophobia	0.2	70%* (31/44)	0.117
Osmophobia	0.2	55% (24/44)	0.028
Nausea	0.06	55% (24/44)	0.587
Allodynia	0.4	70%* (31/44)	0.005
Vertigo	0.4	75%* (33/44)	0.016

### Visit 1: Headache laterality

Headache on the left, right and bilaterally was reported during spontaneous attacks and following nitroglycerin exposure. The agreement analysis for headache laterality is shown in Table 5. There was moderate agreement in nitroglycerin provoking headache with the same laterality dominance as spontaneous attacks.

**Table 5.** Agreement analysis between spontaneous and first NTG-triggered attack and between two NTG-triggered attacks headache laterality.

	Triggered laterality on first NTG exposure (n)			
Spontaneous laterality (*n*)	Left	Right	Bilateral	Totals
Left	10	1	6	17
Right	1	10	0	11
Bilateral	3	3	10	16
Totals				44
Krippendorf's alpha				0.4
Percentage agreement (%)				68%*
	Triggered laterality on second NTG exposure (n)			
Triggered laterality on first NTG exposure (n)	Left	Right	Bilateral	Totals
Left	3	1	1	5
Right	2	5	3	10
Bilateral	5	0	5	10
Totals				25
Krippendorff's alpha				0.2
Percentage agreement (%)				52

### Visit 1: Associated headache symptoms

Associated headache symptoms provoked by nitroglycerin are compared to those during spontaneous attacks in Table 6. The agreement for associated headache symptoms is moderate-to-good for all symptoms apart from osmophobia and nausea.

**Table 6.** Summary of the cranial autonomic symptoms reported following nitroglycerin exposure.

Symptom	Spontaneous attacks (n)	Triggered attack (n)	Number of subjects who had spontaneous symptom successfully triggered with NTG *(n*)	Percentage agreement between spontaneous and first triggered attack (%)
Nasal stuffiness	4	6	2	50
Rhinorrhoea	5	0	0	0
Conjunctival tearing	8	10	3	38
Conjunctival injection	7	5	1	14
Gritty eyes	1	2	0	0
Ptosis	4	0	0	0
Facial oedema	1	0	0	0
Facial sweating	1	0	0	0
Pallor	4	0	0	0
Aural fullness	6	5	3	50
Throat swelling	1	5	1	100
Abnormal taste	2	2	1	50

Between 0–3 (mean 1) cranial autonomic symptoms were triggered in 26 subjects with NTG (49%). Of these 26 subjects, 18 had reported cranial autonomic symptoms associated with spontaneous attacks (69%). Given the relatively small numbers for each symptom group, formal statistical comparison was not performed for these symptoms. A summary of the cranial autonomic symptoms reported spontaneously and during the NTG triggering is summarised in Tables 6 and 7. Cranial autonomic symptoms presented in various phases of the migraine attack triggered by NTG, from the premonitory phase through to following headache resolution in the postdrome.

**Table 7.** Summary of cranial autonomic symptoms reported throughout the triggered migraine attack.

Premonitory phase	Headache phase	Postdrome phase
Conjunctival tearing	Conjunctival tearing	Nasal stuffiness
Conjunctival injection	Conjunctival injection	Throat swelling
Gritty eyes	Nasal stuffiness	Rhinorrhoea
Metallic taste	Gritty eyes	
Throat swelling	Metallic taste	
Nasal stuffiness	Throat swelling	
Aural discomfort	Aural discomfort	
Flushing	Rhinorrhoea	

### Visit 2/3: Premonitory-like symptoms triggered with placebo

**Figure 4.**
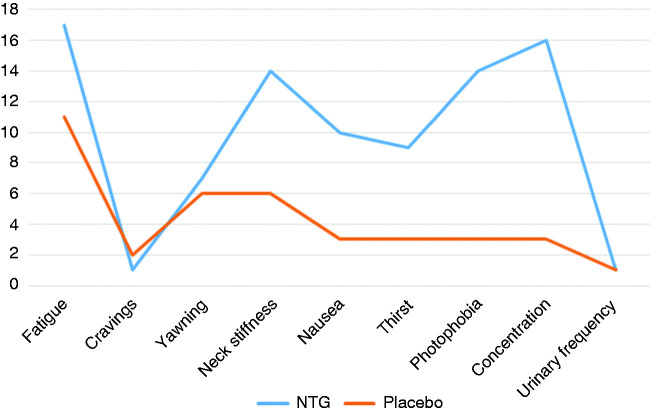
Summary of premonitory symptoms triggered with NTG relative to premonitory-like symptoms triggered with placebo. The y axis represents frequency of symptom reporting.

Paired nitroglycerin and placebo triggering data was available for 21 subjects. At least three premonitory-like symptoms were reported by seven of these when exposed to placebo infusion (33%). The most common symptoms were tiredness (11/21, 52%), yawning (6/21, 29%) and neck stiffness (6/21, 29%). The mean number of symptoms reported with placebo was two (range 0–5). There was a statistically significant difference in the number of symptoms reported following placebo infusion compared to following NTG infusion on visits 2–3 (mean difference four symptoms, 95% confidence interval 3.1–5.7, *t*_20_ = 7.06, *p* < 0.001). Based on randomisation, the number of subjects who received NTG first (n = 11) and placebo first (n = 10) was balanced. There was no significant correlation between the number of placebo-triggered symptoms reported and the baseline headache frequency (Pearson correlation 0.284, *p* = 0.211). Delayed migraine headache was induced by placebo infusion in two subjects. Symptoms provoked by both placebo and NTG are shown in Figure 4.

### Visits 1/2/3: Reliability of symptom triggering

There was a 17% failure to trigger rate at screening, and there was still an increase in the failure to trigger rate of 27% at the next nitroglycerin-triggered visit. For the subjects who had successfully triggered at visit 1 but failed to trigger at the second NTG-triggered visit (n = 9), when the second triggered visit was repeated with consent for some of these (n = 3), they all triggered on the repeat attempt.

Between visits 1 and 2, there were additional subjects who did not continue in the study despite triggering at visit 1 (n = 11), for various reasons including emergence of medical comorbidities and withdrawal of consent.

### Symptom timing

Symptom timing following nitroglycerin exposure on two study visits is summarised in Figure 4. The top row of times represents the screening visit (first nitroglycerin exposure) and the second row the second exposure (visit 2 or 3). The agreement analysis between these timings is shown in Table 8. There was good agreement in the timings to onset of premonitory symptoms and delayed migraine headache following nitroglycerin exposure across two triggered visits.

**Table 8.** Agreement analysis of timings to symptom onset following nitroglycerin exposure on two triggered visits.

*n* = 25	NTG headache visits 1 and 2	Premonitory symptoms visits 1 and 2	Migraine headache visits 1 and 2
Intraclass correlation coefficient	0.440	0.842	0.655
Intraclass correlation *p*-value	0.08	<0.001*	0.006*

### Premonitory symptom phenotype

Agreement analysis for the reliability of premonitory symptom and headache symptom reporting across two triggered visits is shown in Table 9.

**Table 9.** Reliability agreement analysis for the reporting of premonitory and associated headache symptoms on two exposures to nitroglycerin.

Symptom	Cohen's kappa coefficient	Percentage agreement between first and second nitroglycerin- triggered visits (%)	*p*-value
Premonitory			
Tiredness	0.6	88* (23/25)	0.003
Yawning	0.5	76* (19/25)	0.006
Neck stiffness	0.3	72* (18/25)	0.169
Photophobia	0.6	84* (21/25)	0.001
Thirst	0	48 (12/25)	0.973
Nausea	0.3	64* (16/25)	0.06
Mood change	0.4	76* (19/25)	0.02
Concentration difficulty	0.3	76* (19/25)	0.09
Headache			
Photophobia	0	84* (21/25)	0.706
Phonophobia	0	56 (14/25)	0.876
Osmophobia	0.2	84* (21/25)	0.225
Nausea	0	56 (14/25)	0.876
Allodynia	0.5	72* (18/25)	0.021
Vertigo	0	40 (10/25)	0.225

Across two nitroglycerin-triggered visits, agreement for premonitory symptom reporting was moderate to good for all symptoms other than thirst.

### Headache laterality

The agreement analysis for headache laterality triggered by nitroglycerin across two triggered visits is shown in Table 5. There was slight to fair agreement for nitroglycerin triggering the same headache laterality across two triggered visits.

The agreement analysis for the reporting of associated migraine headache symptoms across two nitroglycerin-triggered study visits is shown in Table 9. Across two triggered attacks, phonophobia, nausea and vertigo had the poorest agreement.

## Discussion

Here we report the detailed phenotype of premonitory symptoms, headache and associated headache symptoms provoked by nitroglycerin amongst migraineurs, with additional analyses concerning re-exposure reliability of symptomatology. Triggering rates with nitroglycerin are high, the phenotype, particularly of the premonitory phase, highly reproducible and dissectible from placebo infusion. The premonitory symptom reporting rate was 98%. The existing literature suggests a smaller likelihood of triggering premonitory symptomatology amongst migraineurs (11), but there is likely to be an element of selection bias in this study, as only subjects who reported premonitory symptomatology with their spontaneous attacks were selected. In the future, it would also be informative to study those subjects who do not report spontaneous premonitory symptomatology with nitroglycerin provocation.

As per the study, premonitory symptoms were defined as at least three typical symptoms for the patient – we would not therefore expect at least three of asthenia, dizziness, drowsiness, nausea, vomiting and flushing to present as premonitory symptoms in an individual unless this is what they were used to experiencing prior to a migraine attack. The median time to the development of the premonitory phase was 31 minutes across the subjects in the study, by which time we would have expected NTG effects to have worn off owing to the short half-life and by reference to the vascular imaging data.

Almost all subjects experienced at least one premonitory-like symptom, yet only 83% went on to develop migrainous headache. The development of typical premonitory-like symptomatology in some subjects in the absence of ensuing migraine headache afterwards is interesting. Ideally, a healthy control arm in the study would provide important information about the differences in phenotypic presentation when healthy controls are exposed to nitroglycerin compared to migraineurs, given this has not been before done with a specific view to studying premonitory symptoms. Whilst we have discussed the reasons why the symptoms reported following nitroglycerin in the study are genuine and likely largely unrelated to the infusion: similarity to what subjects would report as similar to their spontaneous migraine attacks and the timing, the issue of what are really drug-induced symptoms would have to involve repeating the study in a healthy control group. This is something that we would like to pursue going forwards using a similar study design.

We report good agreement for many of the common premonitory symptoms reported between spontaneous and triggered attacks. The symptoms with the most difference in subject numbers between spontaneous and triggered attacks were photophobia, which was more commonly triggered than reported spontaneously, and mood change, which was more commonly reported spontaneously. The reasons for this are possibly environmental and related to reporting bias, in that in a bright hospital room where the subject has little else to concentrate on and is asked specifically about a symptom like photophobia, they notice it more, and similarly that, in such a space, mood changes may not be so readily observed, particularly without a collateral witness to corroborate any change and in the absence of any task. When analysing the comparability of the phenotype of spontaneous and triggered symptoms, information was acquired regarding premonitory symptoms based on retrospective recall, whereas for the triggered visit, data was acquired prospectively. This design could have led to suboptimal recognition of premonitory symptoms by subjects during spontaneous attacks, or conversely over-reporting during the triggered attack.

Of the subjects in this study, 83% developed migraine headache following nitroglycerin exposure. With regards to the triggering of migraine headache, our data are consistent with the literature that reports successful triggering rates of 50–83% in migraine with and without aura with the 0.5 mcg/kg/min intravenous nitroglycerin dose (11–13). Headache seemed more likely than not to trigger on the same side as spontaneous attacks, again consistent with the literature (29). There was good agreement for the reporting of photophobia, phonophobia, allodynia and vertigo between spontaneous and triggered attacks. We have previously published the ability of nitroglycerin to trigger cranial allodynia (30). The results for nausea and osmophobia may be related to difficulties assessing sensitivities to smell in the environment of a side room within a research facility. There was significantly less nausea triggered with NTG compared to that reported with spontaneous attacks. This could be related to pain not being allowed to progress and being treated as soon as it reached moderate-severe intensity, and the fact that subjects were not allowed to eat or drink following infusion.

The data demonstrate that NTG is able to trigger vertigo and cranial allodynia, although not quantitatively tested, associated with the migraine attack. In one subject, this manifested in the premonitory phase without any migraine headache, whereas in most it was associated with the headache phase. While nitroglycerin has been used to model allodynia in rodent studies in migraine (31–33), its ability to produce allodynia in humans has not been studied until recently (30), and this finding may make an effective substrate against which to test therapeutic options going forward.

NTG was also able to trigger cranial autonomic symptoms, a novel finding itself. In particular, in some subjects, these were noted in the premonitory phase prior to the onset of headache. Going forwards, systematic questioning about the occurrence of cranial autonomic symptoms and when they occur during the course of the attack may continue to offer valuable insights into the biology of migraine, as it seems that pain is not required for them to ensue. Pre-attack cranial autonomic symptoms have also been noted by a recent study in cluster headache (34).

There is limited reporting on serial triggering rates with NTG. In one study in which 33 of 44 subjects triggered migraine headache with NTG, a re-triggering rate of 97% for headache was reported on second exposure to NTG (11). While in data presented in abstract form, for a study that re-exposed 20 migraineurs to NTG across three visits, 15 of the 20 subjects had delayed migrainous headache following NTG exposure on at least two of the three visits (compared to 0 with placebo), and the headache phenotype was similar to spontaneous, with 89% of attacks responding to usual triptan therapy (35). The results of this triggering study show good reliability between visits for the timing of onset of premonitory and headache symptoms between the first and second NTG-triggered visits.

Despite the small sample size, there was moderate agreement for reporting of the same premonitory symptoms by the same individual across triggered visits for tiredness, yawning, neck stiffness, photophobia and mood change. For headache symptoms, photophobia, osmophobia and allodynia seemed the most consistent symptoms reported across two triggered visits. The reproducibility of allodynia across visits is also an interesting finding, as this symptom seemed to have a moderate level of agreement, both between spontaneous and triggered attacks and between two triggered attacks. The reasons for some of the differences in agreement across visits could be environmental and depend on the level of external stimulation with sound for example, or fluid intake impacting on thirst, as well as the day to day inter-attack variability within individual subjects. The order effect issue should also be noted.

There was no significant agreement between headache laterality triggered with NTG on two triggering visits, although the sample size is relatively small. The reasons for this are unclear; consistency of headache laterality triggering with NTG has only been looked at in one other study (11), where it was reported that laterality across two NTG-triggered visits was reproduced in 93% (52% in this study). This supports the findings regarding other symptoms and the timing data and suggests that there is increased inter-attack variability in the attack phenotype and timing with repeated NTG exposure. The timeline to delayed headache development was generally consistent between NTG-triggered visits, and consistently occurred with a faster timeline compared to spontaneous attacks, with premonitory symptoms occurring only up to 2.5 hours after NTG infusion and headache following shortly after, suggesting that the chance of spontaneous headache occurring during study visits in those with more frequent baseline headache frequency would be unlikely.

### Limitations

Given the study design, all subjects had experienced nitroglycerin infusion prior to the randomised part of the study, and this could have impacted on symptom reporting at each subsequent visit following screening. In addition, given the side effects of nitroglycerin, there are potential issues with maintaining the blind in a study of this kind, as subjects may have become aware of what to expect following the active infusion. However, as can be seen from the placebo data, only in a small number of subjects were similar symptoms experienced following placebo infusion and headache ensued in a small proportion, as would be expected with a migraine placebo response. We did our best to maintain the blind within the remits of the study.

In addition, the subject cohort was heterogeneous and included those with and without aura and those on preventive therapy due to recruitment issues. For the purposes of formal comparison of subjects on and not on preventive treatment, we did not have sufficient statistical power in each group to establish whether there would be a triggering rate difference but would like to pursue this as a question going forwards.

A potential issue when using a pharmacological experimental model, in particular with NTG, is the potential for tolerance with repeated exposure to the substance; that is, the possibility that with serial exposure higher doses may be required to provoke headache or other symptoms, and it is possible that to some extent NTG tolerance could explain some of the variabilities in symptom reporting and headache development in this study. In addition, with each repeated visit, there were fewer subjects involved and therefore a subsequent loss in statistical power, and of course that there are several other environmental and lifestyle factors that were not specifically controlled for in this study and could be modulating the migraine threshold and display of symptomatology across the time in between visits. A short note on terminology is apposite. We have referred to the symptoms occurring before the onset of head pain as the premonitory phase. This has been the term of art to be found in the *Definition of terms* since ICHD-1 (36), which was continued in ICHD-2 (37) and ICHD-3 beta (16). ICHD-3 (6), apparently addlepated and without data, reversed the terms prodrome and premonitory symptoms in verbage. We take this to be an error, and understand its correction is in hand with the Classification Committee. Since the literature has dominantly used the term premonitory symptom over the last 30 years, we advocate its continued use.

## Conclusion

The demonstration of some heterogeneity in migraine symptomatology across triggered visits (intrasubject variability), and the ability of NTG to trigger premonitory symptoms in the absence of ensuing headache, provides experimental support for the theory of a thresholding effect between and within attacks in migraine.

In conclusion, this study provides support for the experimental use of nitroglycerin to study migraine, in particular with regards to capturing experimentally the earliest phase of the attack, with good agreement compared to spontaneous attacks and between attacks with regards to premonitory symptom phenotype and timing, as well as a successful headache trigger rate. The limitations of an order effect, and the loss of statistical power with each repeated study visit, are factors that should be controlled for going forwards. We also plan to perform a similar study amongst healthy controls exposed to nitroglycerin.

## Article highlights

- The nitroglycerin model is an effective means of experimentally provoking premonitory symptoms and headache in a large proportion of migraineurs.
- The agreement in phenotype between spontaneous and triggered attacks is generally good.
- There is reliability both in phenotype and in timing to symptom onset across triggered attacks, with the exception of headache laterality and some associated headache symptoms.
- Cranial autonomic symptoms can ensue following nitroglycerin exposure, even in the absence of pain and at any point during the migraine attack.
- There is a possibility that premonitory symptoms and their relationship to headache could help us further explore an intra-attack threshold within a migraine attack, as well as how a migraine attack starts.
